# Pars Plana Vitrectomy Combined with Focal Endolaser Photocoagulation for Idiopathic Macular Telangiectasia

**DOI:** 10.1155/2014/786578

**Published:** 2014-04-30

**Authors:** Gaku Terauchi, Celso Soiti Matsumoto, Kei Shinoda, Harue Matsumoto, Yutaka Imamura, Emiko Watanabe, Takaaki Kondo, Atsushi Mizota

**Affiliations:** ^1^Department of Ophthalmology, Teikyo University School of Medicine, 2-11-1 Kaga, Itabashi-ku, Tokyo 173-0003, Japan; ^2^Matsumoto Eye Clinic, Tokushima 771-1705, Japan; ^3^Department of Ophthalmology, Teikyo University School of Medicine, University Hospital Mizonokuchi, Kanagawa 213-8507, Japan

## Abstract

*Background*. To report the outcome of pars plana vitrectomy (PPV) combined with intraoperative endolaser focal photocoagulation (PC) on eyes with idiopathic macular telangiectasis (MacTel) type 1. *Methods*. This was a retrospective study of two female patients with MacTel type 1 who were resistant to focal photocoagulation, sub-Tenon triamcinolone injection, and/or antiangiogenic drugs. The best-corrected visual acuity (BCVA) was determined, and fluorescein angiography (FA) and spectral domain optical coherence tomography (SD-OCT) were performed before and after surgery for up to 19 months. *Results*. After surgery, the BCVA gradually improved from 20/100 to 20/20 at 19 months in Case 1 and from 20/50 to 20/13 at 13 months in Case 2. Fluorescein angiography (FA) showed leakage at the late phase, and OCT showed that the cystoid macular edema was resolved and the fovea was considerably thinner postoperatively. *Conclusion*. Patients with MacTel type 1 who are refractory to the other types of treatments can benefit from PPV combined with intraoperative endolaser focal PC with functional and morphological improvements.

## 1. Introduction

Idiopathic juxtafoveal macular telangiectasia (MacTel) is characterized by vascular anomalies affecting the macular capillary network. It was first described by Gass and Oyakawa [1] and Gass and Blodi [2] and named idiopathic juxtafoveolar retinal telangiectasis (IJRT). It was recently renamed macular telangiectasis (MacTel) by Yannuzzi et al. [3]. There are two types of MacTel: type 1 with aneurysmal telangiectasia and type 2 with parafoveal telangiectasia. MacTel type 1 or unilateral parafoveal telangiectasis (Group 1B IJRT) typically occurs in one eye of relative young men. The temporal half of the macula is involved by the telangiectasis, and the macular oedema and hard exudates lead to vision reduction. No treatment has been established although some encouraging effects have been obtained by argon laser photocoagulation (PC) [4, 5], intravitreal or sub-Tenon's capsule injection of triamcinolone acetonide (IVTA or STTA) [5–7], or intravitreal bevacizumab (IVR) or ranibizumab (IVB) injections [8–10] in small case series.

We present two patients with MacTel type 1 who were refractory to photocoagulation (PC), STTA, and IVB but responded to pars plana vitrectomy (PPV) combined with intraoperative endolaser focal PC.

## 2. Materials and Methods

This was a retrospective study of two eyes of two patients with MacTel type 1 who did not respond to focal PC delivered by an integrated slit lamp, to STTA, and/or to IVB. After discussing the possible treatment options including repetition of earlier treatments, an informed consent was obtained for our technique of PPV combined with intraoperative endolaser focal PC. Both patients underwent PPV combined with endolaser focal PC during the surgery. The diagnosis of MacTel type 1 was based on the fundus examination, FA, and OCT after the exclusion of neovascular maculopathy, secondary macular telangiectasia, and diabetes. Both eyes had cystoid macular oedema (CME) and showed a prompt filling of both the superficial and deep capillary networks of the telangiectatic vessels. There was also late intraretinal staining by fluorescein. The follow-up period was 19 months for Case 1 and 11 months for Case 2.

The ocular examinations included measurements of the BCVA, ophthalmoscopy, fluorescein angiography (FA), and spectral domain optical coherence tomography (SD-OCT). Serial SD-OCT B-scan images were obtained with the Cirrus HD-OCT (Carl Zeiss Meditec, Dublin, CA, USA). The foveal thickness (FT) was measured as the distance between the internal limiting membrane and inner border of the retinal pigment epithelium at the foveal centre with the computer-based caliper built into the OCT system. The vertical and horizontal B-scan images across the fovea were used to determine the foveal thickness.

## 3. Case Reports

### 3.1. Patient 1

**Figure 1:**
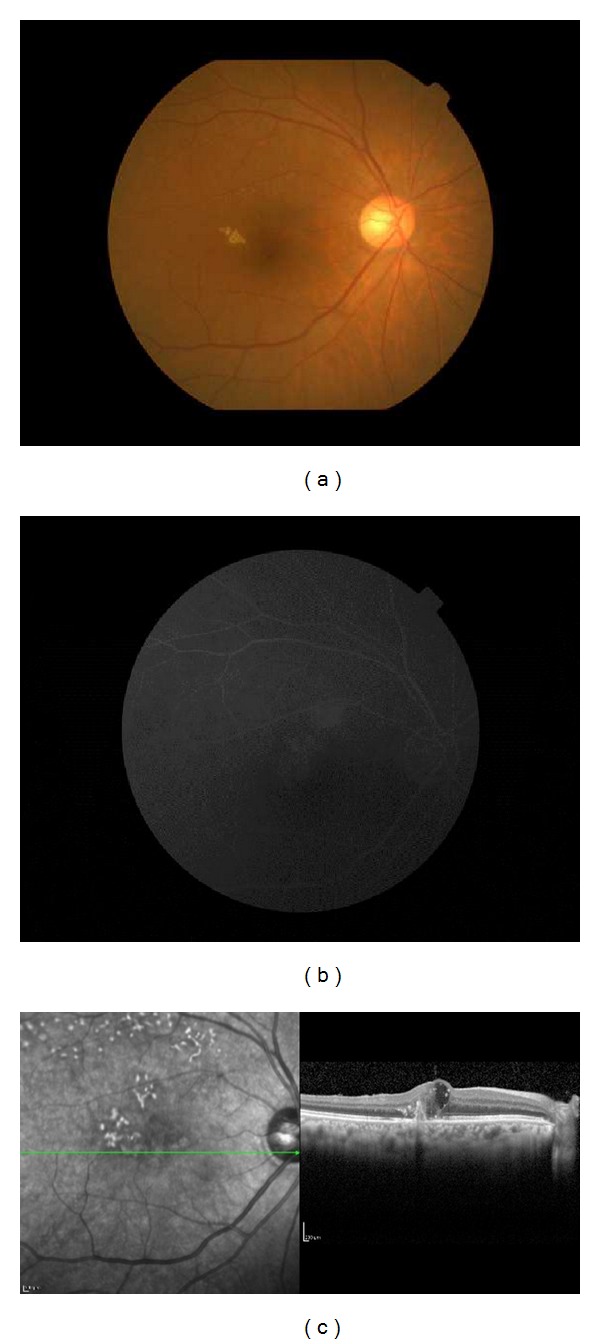
Finding of the right eye of Patient 1 with idiopathic macular telangiectasis (MacTel) type 1 on her first visit. Her best-corrected visual acuity (BCVA) was 20/100. (a) Fundus photograph showing hard exudates associated with telangiectasia temporal to the fovea. (b) Fluorescein angiogram showing strong fluorescein leakage in the late phase. (c) Optical coherence tomographic (OCT) image showing cystoid macular edema in the area surrounding the leakage.

A 79-year-old woman complained of blurred vision in her right eye and came to our clinic. Her BCVA was 20/100 OD and 20/25 OS. FA showed telangiectasia temporal to the fovea with pronounced fluorescein leakage in the late phase in the area of the telangiectasia. OCT showed cystoid macular edema (CME) in the area surrounding the leakage (Figure 1). The right eye was diagnosed with MacTel type 1 and received STTA, IVB twice, and focal PC through a slit lamp. These treatments failed to decrease the leakage on FA and resolve the CME. The BCVA was not improved.

After discussing the treatment options, the patient gave us an informed consent for PPV with a 25-gauge trocar system combined with the endolaser focal PC on the right eye. After core vitrectomy, a posterior vitreous detachment was created by suction through the vitreous cutter. The internal limiting membrane was made more visible with triamcinolone acetonide particle (Maqaid), and it was grasped and peeled with a microforceps. Then, focal PC was performed on the fluorescein leakage points with a 25-gauge endolaser probe and 100 to 120 mW power so that the focal retinal edema was treated.

**Figure 2:**
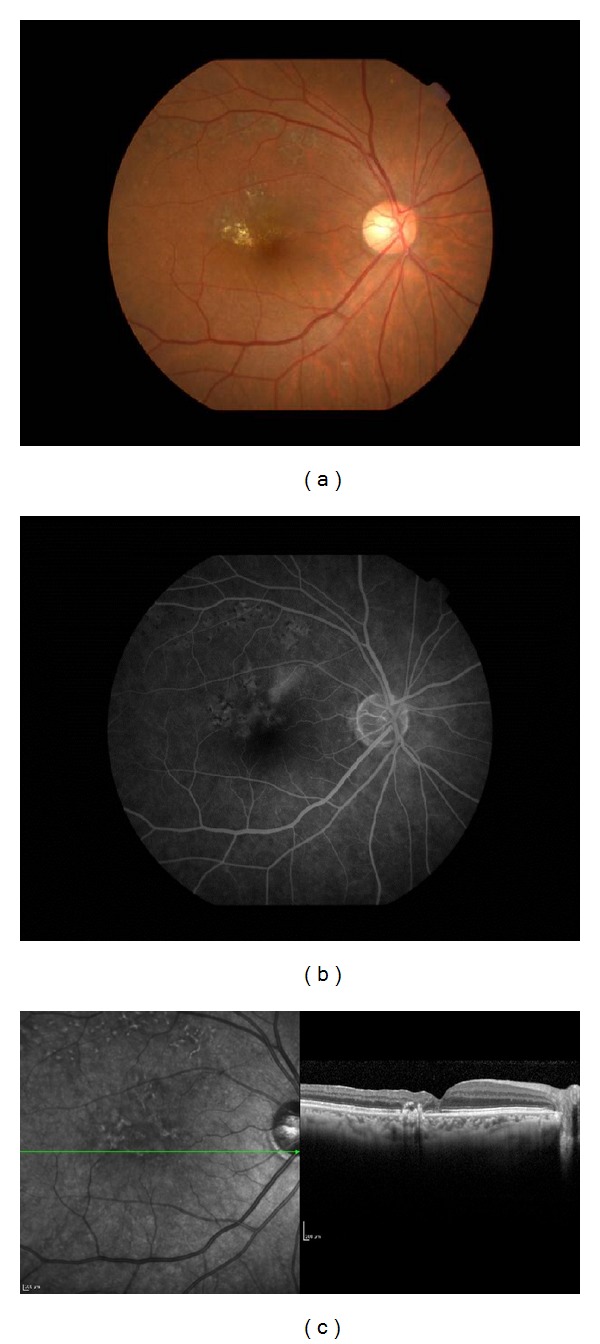
Findings of the right eye of Case 1 taken 3 months after surgery. The BCVA has improved to 20/25. (a) Fundus photograph showing localized area of scars from the laser photocoagulation temporal to the fovea. (b) Fluorescein angiogram showing the absence of fluorescein leakage in the late phase. (c) Optical coherence tomographic image showing an absence of cystoid macular edema and regained foveal pit.

**Figure 3:**
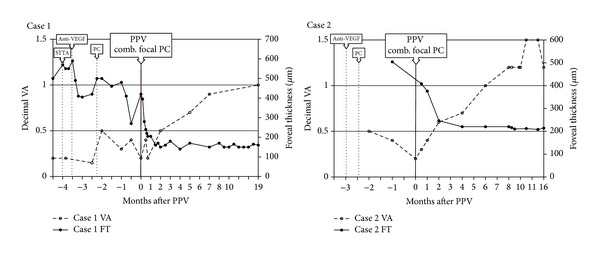
Clinical course of the affected eyes in two cases of MacTel type 1. In Case 1, the visual acuity improved to 20/20 and foveal thickness was reduced to 140 *μ*m at 19 months after surgery. In Case 2, the visual acuity improved to 20/13 and foveal thickness to 208 um at 13 months after surgery.

After that, the CME decreased and the BCVA improved gradually to 20/25 in 3 months. The leakage of fluorescein was not present, the CME could not be detected in the OCT images, and the foveal thickness decreased from 420 to 140 *μ*m (Figure 2). During the 19-month follow-up period, the BCVA and the CME progressively improved (Figure 3).

### 3.2. Patient 2

A 69-year-old woman with no relevant medical history presented with decreased vision in her left eye of 1-week duration. She had been diagnosed with macular oedema associated with MacTel type 1 and underwent IVB and focal PC in a private clinic. The treatments were not effective, and she was referred to us two months later.

**Figure 4:**
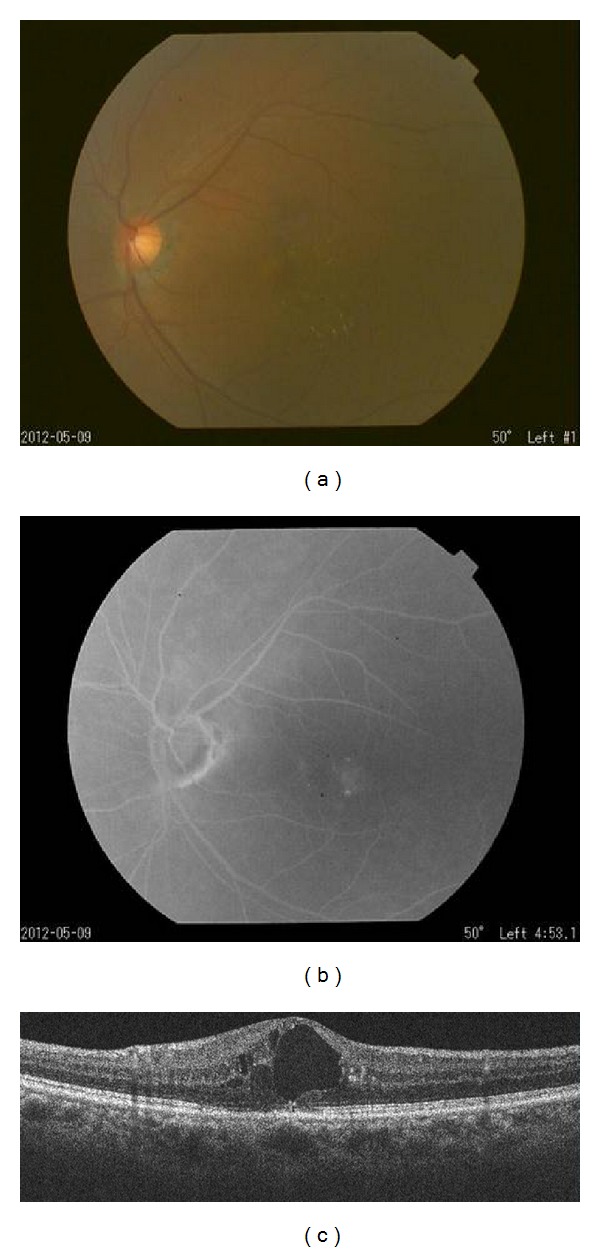
Findings of the left eye at the first visit of Case 2. The BCVA was 20/50. (a) Fundus photograph showed hard exudates associated with telangiectasia inferior temporal to the fovea. (b) Fluorescein angiogram showing fluorescein leakage in a circular pattern in the late phase. (c) Optical coherence tomographic image showed cystoid macular edema at the macula surrounded by circularly arranged fluorescein leakages.

Our examination showed that her BCVA was 20/20 OD and 20/50 OS. FA revealed ectatic capillaries temporal to the fovea with leakage in the late phase in both eyes but especially in the left eye. SD-OCT showed severe CME in the left eye (Figure 4). She was diagnosed with MacTel type 1 and underwent PPV with intraoperative endolaser focal PC as in Patient 1.

**Figure 5:**
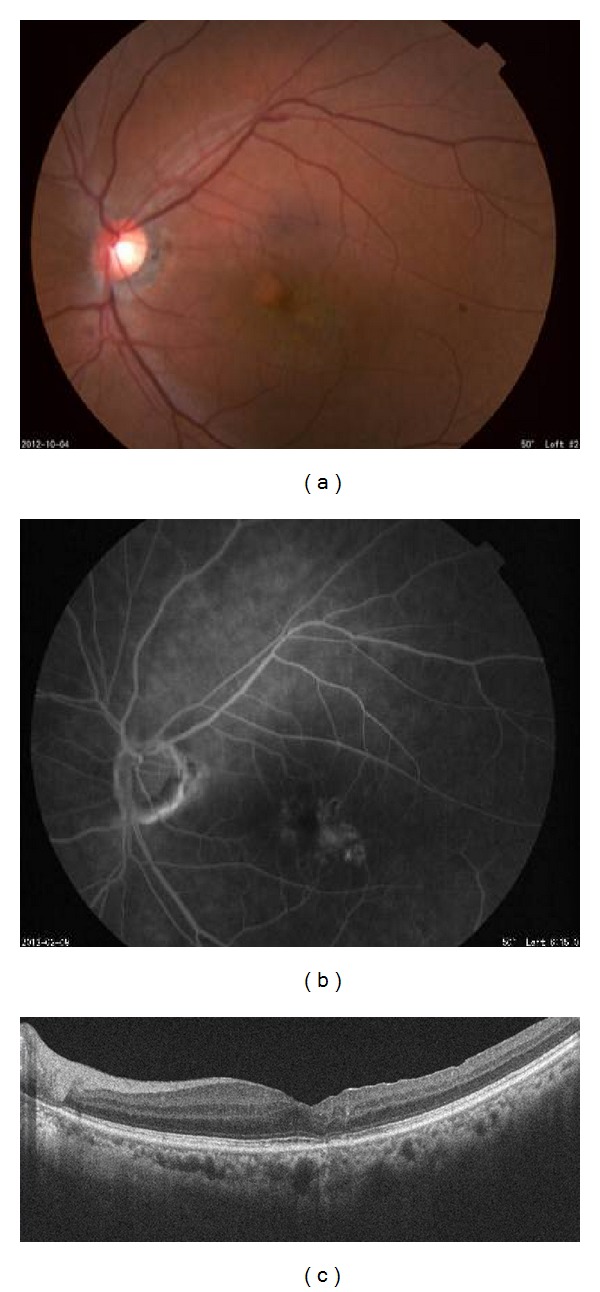
Fundus appearance of the left eye of Case 2 six months after surgery. Visual acuity has improved to 20/13. (a) Fundus photograph showed localized area of scarring by laser photocoagulation inferior-temporal to the fovea. (b) Fluorescein angiogram showing the disappearance of fluorescein leakage in the late phase. (c) Optical coherence tomographic image showing the absence of cystoid macular edema and restored foveal contour.

After that, the CME decreased and her BCVA improved gradually to 20/13 in 6 months. The leakage of fluorescein was not present, and the CME in the OCT images was not detected. The FT decreased from 512 *μ*m to 200 *μ*m (Figure 5). The clinical course of the left eye is showed in Figure 3. Nine months later, the right eye developed CME, but the BCVA remained at 20/20.

## 4. Discussion

Our results showed that PPV with endolaser focal PC can improve the BCVA and reduce the CME in patients with MacTel type 1. Our cases had not responded to focal PC through an integrated slit-lamp system, STTA, and/or antiangiogenic drugs, but after PPV with endolaser focal PC, the vision and CME improved. These findings strongly suggest a causal relationship between the treatment and the improvements.

Several treatments have been reported to be effective for MacTel, especially for type 2 [7, 8, 11], and there are few reports on the treatment of MacTel type 1 [4, 8–10]. IVTA or STTA has been reported to be effective in some cases [3–7] because steroids are anti-inflammatory and might maintain the blood-retina barrier. Recently, antiangiogenic drugs such as bevacizumab or ranibizumab have been reported to be effective in some cases of MacTel type 1 [8–10]. Antiangiogenic drugs are known to reduce neovascularization and oedema; however the follow-up times in those reports were relatively short and some cases had recurrences. Therefore, the efficacy of those therapies has still not been definitively determined.

At present, there is no consensus regarding the treatment of MacTel. Our two patients had no or only limited improvement clinically and angiographically after PC, STTA, and/or antiangiogenic therapy. Thus, we believed that intraoperative endolaser focal PC may be more effective because it allows for better accuracy in treating the lesions than through an integrated slit-lamp delivery system. Focal PC through a slit lamp has several disadvantages. The site of the lesion can be easily affected by micromotions of the eye, the use of a joystick and manipulation of the contact lens require considerable technique and experience, reflected light from the contact lens can reduce the visibility of the macular region, and the endolaser beam can be delivered at different angles which can reduce the energy to the retinal pigment epithelium (RPE) over the fovea. The RPE is located in the outer layer and microaneurysm is in the inner layer, and the laser beam that is delivered obliquely from the inner and central side arrives relatively peripheral to the outer layer. This can prevent damage to the RPE. And finally, the endolaser procedure is not influenced by an opaque media, and the intravitreal laser probe can be brought very close to the retinal surface.

However, there are also drawbacks to the endolaser photocoagulation such as the difficulty for repeated treatments because of the risks associated with intraocular surgery.

There are several factors that may have played a role in improving the macular edema after PPV with endolaser focal PC. The removal of the vitreous and/or ILM may have reduced the level of pathological cytokines or chemical mediators adjacent to the telangiectasia. There are several reports showing that ILM peeling is effective treatment for macular edema secondary to diabetic retinopathy (DME) [12, 13] and retinal vein occlusion (RVO) [14]. Although the mechanism of the ILM peeling has not been fully understood, it might have contributed to the successful outcome. The intraoperative use of TA may have similar effect as STTA or IVTA although its use was only transient. The effectiveness of PPV alone can be assessed if intraoperative PC was not done. But the therapeutic protocol did not allow it. In addition, Sigler et al. reported that PPV was not effective against nonproliferative idiopathic MacTel type 2 [15]. MacTel type 1 is mainly exudative and nonfamilial, while type 2 is primarily nonexudative, obstructive, and occasionally familial. This may explain the differences of our results from the results of Sigler et al. In addition, some cases of MacTel type 1 respond well to antiangiogenic drugs but not type 2.

There are some limitations in our study. This was a retrospective study of only 2 patients. In addition, the follow-up period was short, and there were no controls. However, we believe that PPV with endolaser focal PC is effective and should be considered as an optional treatment for selected cases of MacTel type 1 especially in refractory cases. These treatment protocols should lead to an improvement in both the BCVA and macular edema.

In conclusion, we have experienced two patients with MacTel type 1 who were refractory to photocoagulation (PC), STTA, and IVB but responded to pars plana vitrectomy (PPV) combined with intraoperative endolaser focal PC.

Although further investigations are needed to elucidate the rationale and to establish its indication, we think a stepwise approach to the management of the disease with the use of surgical management can be considered when conventional treatment fails.
